# Reliability of the Fenton growth curve method in predicting birth weight in newborns born at or before 36 weeks gestational age

**DOI:** 10.3389/fped.2025.1482823

**Published:** 2025-02-13

**Authors:** Sara Gungor, Wei Hou, Joseph Decristofaro, Echezona T. Maduekwe

**Affiliations:** ^1^ Department of Pediatrics, Neonatology, Stony Brook Children’s Hospital, Stony Brook, NY, United States; ^2^ Family, Population and Preventive Medicine, Stony Brook Children’s Hospital, Stony Brook, NY, United States

**Keywords:** neonate, weight percentile, resuscitation, fetal ultrasound, Fenton curve

## Abstract

**Objectives:**

The study aimed to assess the reliability of the Fenton curve superimposed with fetal weight percentile in predicting the birth weight of infants born ≤36 weeks gestational age.

**Study design:**

This prospective observational study, conducted from December 2018 to May 2019, examined infants born ≤36 weeks gestational age. The Fenton curve was used to extrapolate the fetal growth percentile (Fenton curve method) and predict the birth weight of the infants on the day of delivery. The study excluded infants who were large or small for gestational age and those diagnosed with congenital anomalies or Hydrops fetalis. The Fenton curve method was used to predict the actual birth weight of 65 infants born at Stony Brook University Hospital and was compared with their actual birth weight using a two-sample *t*-test and Bland–Altman test.

**Results:**

The study enrolled 65 infants, including 37 females, 2 Asians, 6 Blacks, 17 Hispanics, and 40 whites, with a mean gestational age of 32.1 ± 3.6 weeks and a mean actual birth weight of 1,860 ± 677 grams. The results showed no significant difference between the predicted and actual birth weight (*p* = 0.17), with a median difference of 32.2 grams between the predicted and actual weight.

**Conclusion:**

The study found that the Fenton curve method reliably predicted the birth weight of infants born ≤36 weeks of gestational age. The results indicate that in normally grown fetuses, healthcare providers in the delivery room could potentially use Fenton curve predicted birth weight for drug dosage and equipment sizing in scenarios where obtaining actual birth weight is not possible. Nonetheless, additional analysis in larger cohorts is needed before this method can be universally adopted.

## Introduction

Infants who experience emergencies right after birth may need life-saving interventions, including intubation and drug administration. Unlike adults, emergency drug dosages and infant equipment sizes are determined based on weight. Therefore, it is crucial to accurately measure or estimate an infant's weight for effective management during emergency neonatal resuscitation. About 10% of newborns require some assistance to start breathing after birth, and less than 1% require extensive resuscitation, including intubation and medications (1).

Accurately estimating birth weight is crucial for life-saving interventions, but it is usually not possible to measure the actual weight during extensive resuscitation of a sick newborn. Previous studies have shown that medical errors, such as miscalculated pediatric medication dosages, are common, with incorrect estimation of a patient's weight being the most frequently reported error (2–4). To minimize the consequences of medication errors, especially when it is not always feasible to measure an infant's or child's weight, the Broselow color-coded length-based tape measure is recommended by the Pediatric Advanced Life Support (5, 6). While two previous studies showed that the Broselow tape accurately estimates weight in children (7, 8), Nieman et al. concluded that it under-dosed 30% of the children (9). These studies included neonates weighing at least 3,000 grams. Therefore, the Broselow tape is a standard tool in the pediatric crash cart in the emergency room and pediatric intensive care unit. Still, it is not part of the neonatal resuscitation tool.

Ultrasound estimation of fetal weight is essential for managing pregnancies, providing crucial information for determining the timing and method of delivery for pregnant women (10–12). However, caregivers' use of ultrasound-estimated weight at delivery is limited by the discrepancy between the ultrasound date and the delivery date, making birth weight estimation challenging on the day of birth. Due to the lack of a reliable method to predict birth weight on the day of delivery, providers often resort to “guestimates” when accurate weight measurement during neonatal resuscitation is not possible. While visual guestimates are common during newborn resuscitation, their reliability and accuracy are not known. The potential for these “guestimates” to result in severe consequences, such as incorrect drug dosing or equipment size selection, which are critical for a patient's survival, highlights the urgent need for a more objective method of birth weight estimation.

The Fenton growth chart is a widely used birth-size reference chart created using data from several cross-sectional population studies (13, 14). Having sonographic fetal weight percentile and Fenton curves available before most deliveries is essential for predicting birth weight. Hence, assessing the reliability of the fetal weight percentile superimposed on the Fenton curve is crucial. This method of predicting birth weight has the potential to significantly improve the accuracy of resuscitation decisions, leading to better patient outcomes. Therefore, this study aims to evaluate the reliability of the Fenton curve method in predicting the birth weight of newborns. Additionally, the study examines the impact of the timing of the ultrasound on the reliability of the Fenton curve method in predicting birth weight. We hypothesized that the Fenton curve method would be reliable in estimating birth weight at delivery.

## Materials and methods

In this prospective, observational study conducted at Stony Brook Children's Hospital from December 2018 through May 2019, we aimed to predict the birth weight of neonates born ≤36 weeks gestational age. We excluded infants with fetal growth restriction (<10th percentile for gestational age on the fetal ultrasound), congenital anomalies such as gastroschisis and neural tube defects, hydrops fetalis, large for gestational age fetus (>90th percentile for gestational age on the fetal ultrasound), and infants whose parents declined consent.

On the day of delivery, we overlay the estimated fetal weight percentile from the latest ultrasound onto the modified Fenton curve for the newborn's gestational age to predict birth weight. The obstetricians used the Hadlock method (15), which considers the fetal head circumference, abdominal circumference, and femur length, for fetal weight and weight percentile estimation. We applied the modified Fenton curve using the PediTools® electronic growth chart calculator (13, 16). We recorded each enrolled patient's predicted (fetal weight percentile determined weight on the Fenton curve) and actual weights (measured weight at delivery). Additionally, we collected maternal and neonatal demographic data from the electronic medical record. The newborn's actual weight was obtained using the digital weighing scale integrated into the radiant warmer. Following our hospital's routine, we used the gestational age provided by the obstetrician (based on the mother's last menstrual period) as the newborn's gestational age at birth.

We aimed to include 65 infants to identify a difference of ≥10% between the predicted and actual birth weights, with an estimated error of 5%. We compared the mean and standard deviations of the predicted weights with the actual birth weights and utilized a pairwise *t*-test for data analysis. Additionally, we conducted a Bland and Altman analysis (17) to calculate the mean difference between actual and predicted birth weight with a 95% confidence interval (CI) and constructed limits of agreement at ±1.96 SD, where 95% of the differences between actual and predicted birth weight are found. We established that a weight difference of 500 grams (0.5 Kg) would be clinically relevant and expected the observed limits of agreement to fall within this range.

The SUNY Stony Brook Institution Review Board approved the study with informed consent. We analyzed the data using the SAS v9.4 (SAS Institute, Cary, NC) for statistical analysis and considered differences with *p* values <0.05 as statistically significant.

## Results

**Figure 1:**
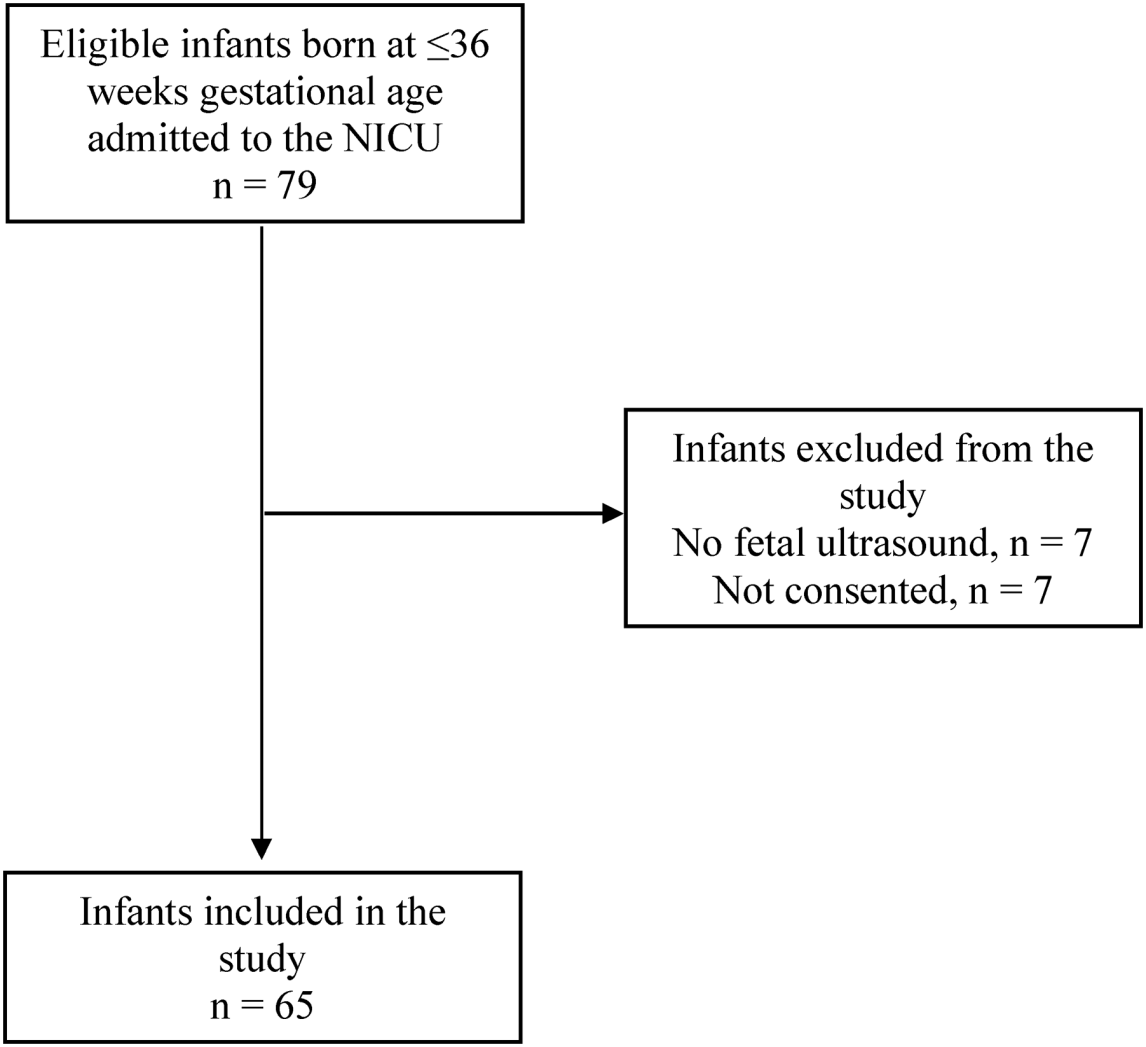
Flow diagram of study subjects.

Seventy-nine infants were initially eligible for the study, but ultimately, only 65 were enrolled (as shown in Figure 1). Among the 65 infants, 37 (57%) were females. The majority (62%, 40/65) were Caucasians, while 2 (3%) were Asian, 6 (9%) were black, and 17 (26%) were Hispanic. The median gestational age was 34 weeks (24.1–35.6 weeks). Thirteen (20%) infants were born before 28 weeks, 11 (17%) were born between 28 weeks and 32 weeks, 25 (38%) were born between 32.1 weeks and 35 weeks, and 16 (25%) were born after 35 weeks of gestation. Cesarean section was the predominant delivery method, accounting for 69% (45/65) of the cases. The majority (48%, 31/65) fell between the 50th and 74th percentiles on the fetal ultrasound (refer to Table 1).

**Table 1:** Demographic characteristics of study infants (*n* = 65).

Variable	Level	Number
Gender, *n* (%)	F	37 (57)
	M	28 (43)
Race, *n* (%)	Asian	2 (3)
	Black	6 (9)
	Hispanic	17 926)
	White	40 (62)
Gestational age in weeks (mean ± SD)	32.1 (3.6)	65 (100)
Delivery, *n* (%)	C-section	45 (69)
	Vaginal	20 (31)
Fetal weight percentiles, *n* (%)	10–24th	6 (9)
	25–49th	24 (37)
	50–74th	31 (48)
	75–89th	4 (6)
Gestational ages at fetal ultrasound in weeks, *n* (%)	<28	13 (20)
	28–32	11 (17)
	32.1–35	25 (38)
	>35	16 (25)

Data are *n* (%), mean ± SD. C-section, caesarean section.

### Comparing the Fenton curve method predicted birth to the actual birth weight

**Figure 2:**
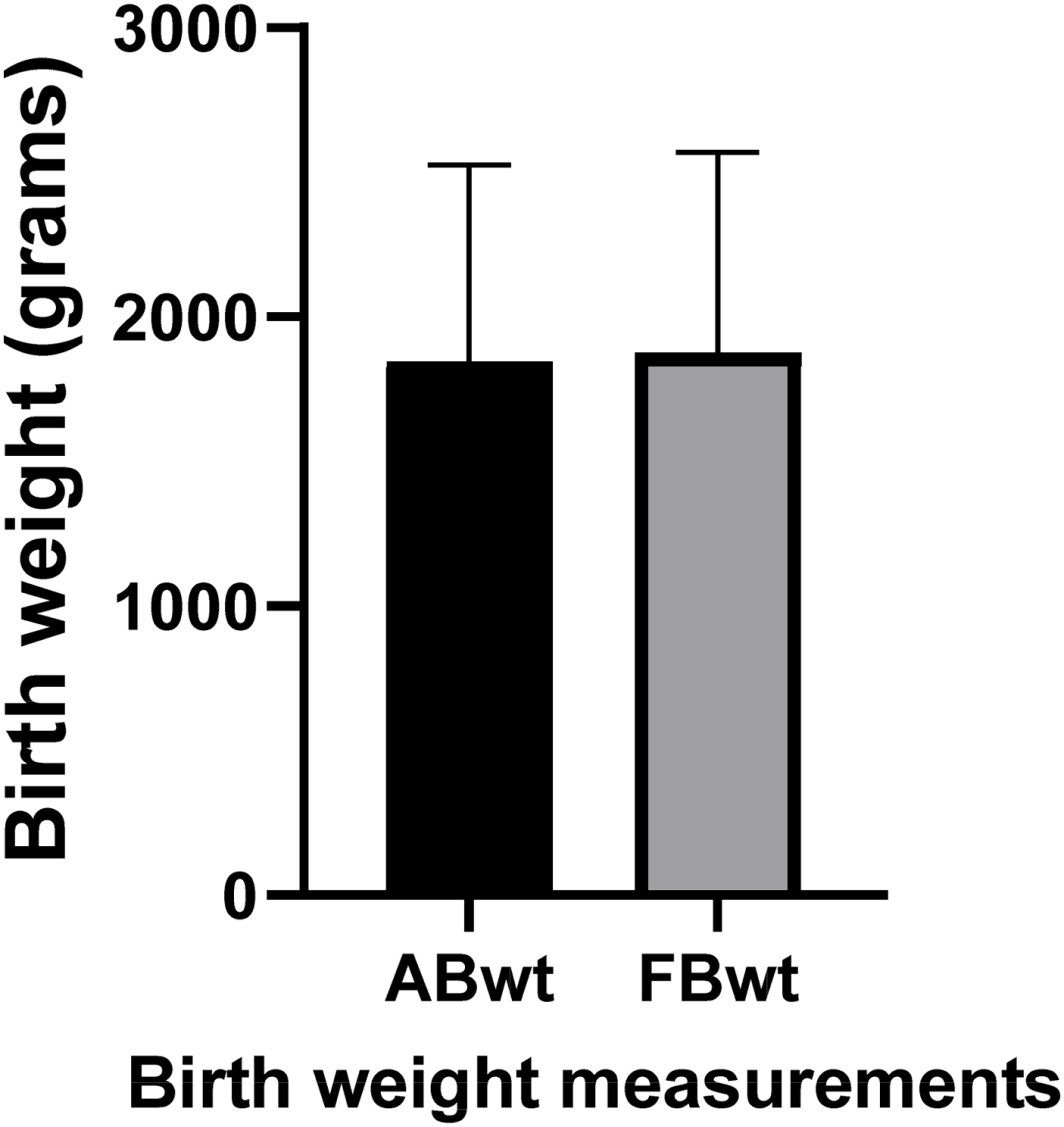
Comparison of actual and predicted birth weight. A paired *t*-test comparison of the actual and predicted birth weights shows no statistically significant difference in weights (*p*-value = 0.17). ABwt, actual Birth weight; FBwt, Fenton curve predicted birth weight.

**Figure 3:**
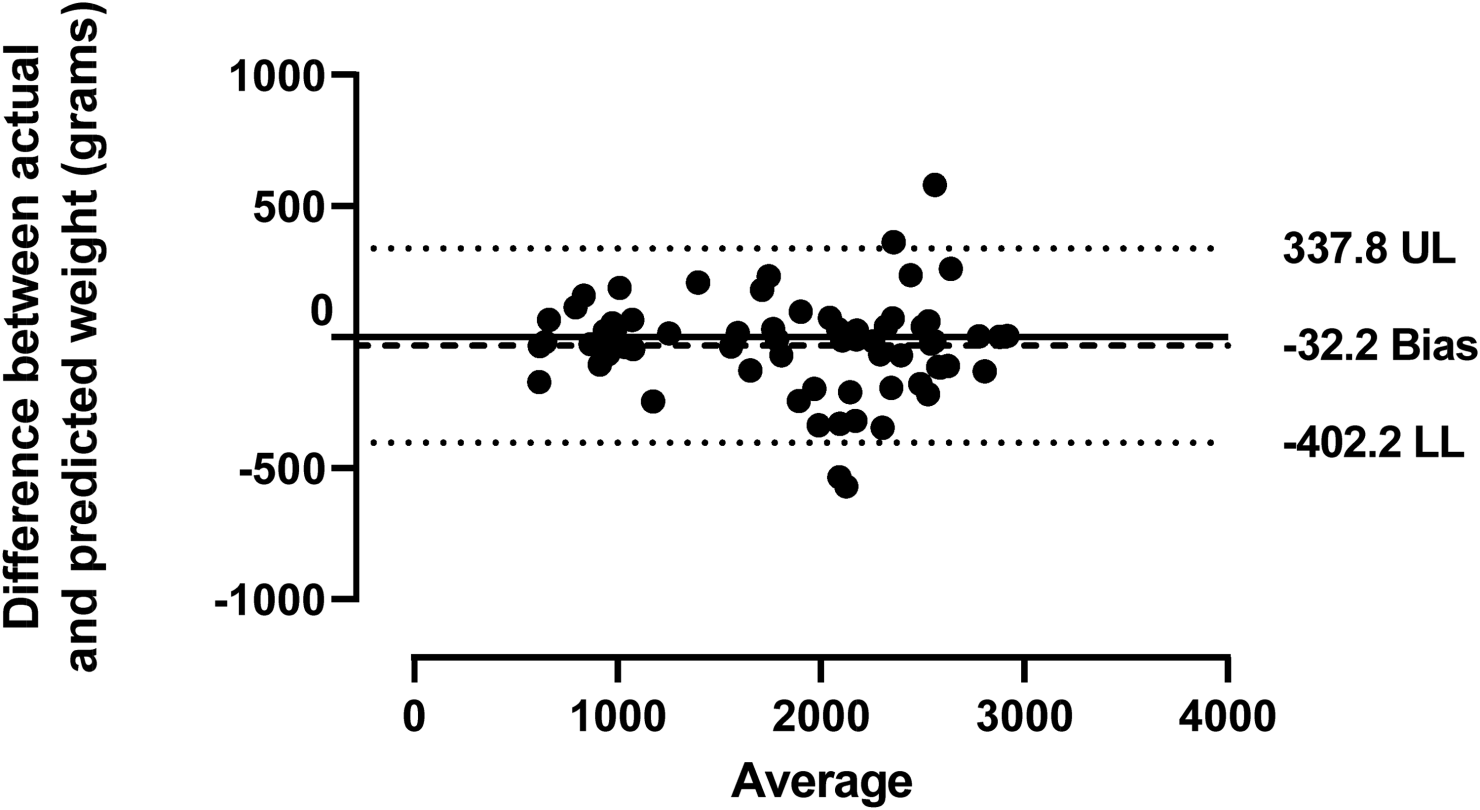
Agreement between the actual and predicted birth weights. Bland and Altman's plot shows the agreement between the actual and predicted birth weights using linear regression and a 95% confidence interval. The solid line represents the mean, while the lighter single dotted lines indicate the limits of agreement at ±1.96 SD. UL, upper limit; LL, lower limit.

The average actual birth weight of the infants was 1,845 ± 672 grams, which was not significantly different from the mean predicted birth weight, 1,877 ± 684 grams (*p*-value 0.17, Figure 2). The Bland and Altman analysis showed slight variations between birth weights, with a mean difference of 32.2 grams and limits of agreement of −402.2 to 337.8 (95% CI, −14.57 to +78.97), as shown in Figure 3. This minor difference was consistently observed across the entire range of measurement values. The Fenton curve method slightly underestimated the birth weight for infants born <28 weeks gestation with a mean percentage error of 1.3% (mean difference of 10.9, SEM 28.6 grams) and between 28 and 32 weeks gestation with a mean percentage error of 0.6% (mean difference of 7.6, SEM 38.2 grams). However, it slightly overestimated the birth weight for infants born from 32.1 to 35 weeks gestation with a mean percentage error of 1.7% (mean difference of −36.2, SEM 48.2 grams) and >35 weeks gestation, mean percentage error of 3.6% (mean difference of −88.4, SEM 46 grams). These differences were not statistically significant, with an overall mean percentage error of 1.7% and a *p*-value of 0.17 (Table 2).

**Table 2:** Comparing actual and predicted weight at different gestational ages (*n* = 65).

Gestational age (weeks)	Number (%)	ABwt (mean ± SD) grams	FBwt (mean ± SD) grams	Mean difference ± SEM, grams	Mean percentage error, %	*P* value
<28	13 (20)	845 ± 178.6	834 ± 152.4	10.9 ± 28.6	−1.3	0.71
28–32	11 (17)	1,385 ± 309.2	1,377 ± 286.3	7.6 ± 38.2	−0.6	0.85
32.1–35	25 (38)	2,169 ± 324.5	2,205 ± 271.8	−36.2 ± 48.2	1.7	0.36
>35	16 (25)	2,469 ± 294.5	2,558 ± 202.8	−88.4 ± 46	3.6	0.07
Overall (24.1–35.6)	65 (100)	1,845 ± 677.2	1,877 ± 690.1	−32.2 ± 23.4	1.7	0.17

Data are *n* (%), mean ± SD, mean ± SEM. ABwt, actual birth weight; FBwt, Fenton curve predicted birth weight.

### Effect of interval from ultrasound to delivery on the reliability of birth weight prediction

In our study, we found no significant difference between the predicted and actual birth weights, regardless of whether the interval between the ultrasound scan and delivery was ≤2 weeks (95% CI, −5.12 to 102, *p*-value 0.08) or >2 weeks (95% CI, −98.8 to 94.3, *p*-value 0.96). However, the mean difference between the predicted and actual birth weight was negligible with the ultrasound scans performed >2 weeks (2.24 grams), compared to those performed ≤2 weeks before delivery (48.6 grams), as presented in Table 3.

**Table 3:** Comparing actual and predicted birth weight at different fetal ultrasound time points (*n* = 65).

Duration of ultrasound before delivery	Interval from ultrasound to delivery (wks. ± SD)	ABwt (mean ± SD), grams	FBwt (mean ± SD), grams	Mean difference	*P* value
≤2 weeks	1.0 ± 0.5	1,692 ± 633	1,741 ± 667	−48.6 (95% CI, −5.12 to102)	0.08
>2 weeks	6.6 ± 5.1	2,166 ± 669	2,164 ± 663	2.24 (95% CI, −98.8 to 94.3)	0.96

Data are mean ± SD. Wks., Weeks; ABwt, actual birth weight; FBwt, Fenton curve predicted birth weight.

## Discussion

In our study involving 65 infants born between 24.1 and 35.6 weeks gestational age, we found that using the Fenton curve method reliably predicted birth weight on the day of delivery. We also observed that the interval in weeks from ultrasound to delivery did not significantly affect the reliability of predicted birth weight. These findings suggest that using the Fenton curve method can help in determining drug dosages and selecting appropriate equipment sizes during the emergency resuscitation of a newborn.

To our knowledge, this is the first study to use superimposition of weight percentile from the fetal ultrasound on the Fenton curve to predict the newborn weight at birth. Previous studies have focused on the accuracy of ultrasound estimation of fetal weight for birth weight prediction (10–12). While the ultrasound estimation of fetal weight is essential in identifying high-risk populations for the obstetrician, its use during emergency newborn resuscitation is limited by the interval between the gestational age at the ultrasonographic scan and the gestational age at delivery. Although it relies on fetal ultrasound, this Fenton curve method eliminates this limitation.

In our study, we found that the predicted weight is not significantly different from the actual weight in our group of infants born between 24.1 and 35.6 weeks of gestational age. However, it is essential to note that the Fenton curve slightly underestimated the birth weight of infants born ≤32 weeks of gestation and overestimated the birth weight of infants born >32 weeks. The reason for this observed variance is unknown but may be related to the inaccuracy of the ultrasound. Nonetheless, a slight underestimation of the birth weight of infants by the Fenton curve method may be clinically beneficial in reducing the risk of overdosing, especially in those born ≤32 weeks gestation (percentage error of mean ≤1.3%). Moreover, accurately predicting birth weight within 5% of actual birth weight is critical for minimizing random error (11, 18).

Furthermore, we found no significant difference between the predicted and actual birth weights, regardless of the interval between the ultrasound scan and delivery. However, we observed that the average difference between the actual and predicted birth weight was almost negligible when the ultrasound was done >2 weeks before delivery compared to when it was done ≤2 weeks before delivery (2.24 g vs. 48.6 g). This finding suggests that using the weight percentile from an ultrasound performed at least 2 weeks before delivery provides a more precise prediction of birth weight than using the weight percentile from an ultrasound performed ≤2 weeks before delivery. This finding is valuable as it reduces the necessity for ultrasound close to delivery to determine birth weight.

Our current study has several limitations that primary caregivers should consider when applying the findings to clinical practice. First, our research is limited to appropriate-for-gestational-age fetuses/newborns, excluding extreme sizes such as small or large-for-gestational-age fetuses/newborns. This exclusion was due to our institution's lack of specific weight percentiles on fetal ultrasound. Weight percentiles on the fetal ultrasound were recorded as >90th percentile for large fetuses and <10th percentile for small fetuses, making it challenging to accurately match a non-specific weight percentile to the Fenton curve. Future studies, including small and large gestational-age infants, will help address this limitation. Second, our study focused on weight percentiles from a fetal ultrasound using the Hadlock formula. Therefore, our findings may not directly relate to weight percentiles determined using formulae other than Hadlock's. This highlights the need for a prospective multi-centered study to comprehensively evaluate the impact of different ultrasonographic formulae on birth weight prediction. Lastly, our study is limited to preterm and late preterm infants (<37 weeks gestation) and cannot be applied to infants delivered ≥37 weeks gestation. Thus, fetal weight percentiles need to be tested on a curve designated for term infants, such as the Centers for Disease Control (CDC) curve. This may be achieved by conducting a prospective study using the fetal weight percentile on the CDC growth curve.

The strengths of this study lie in its ability to predict birth weight using fetal weight percentile and the Fenton growth curve commonly employed in clinical practice. This approach enables us to generate a more generalizable evaluation of the reliability of our new method for predicting birth weight. Additionally, the obstetricians were unaware of the weight percentile superimposition on the Fenton curve, thus minimizing unintentional bias.

## Conclusion

When superimposed on the Fenton curve, the weight percentile on the fetal ultrasound may be reliable in predicting the birth weight of newborns of appropriate gestational age. Healthcare providers can utilize this estimation method when the birth weight is unknown, which helps determine medication dosage and select the right equipment size during emergency resuscitation. Implementing this approach could improve the accuracy of resuscitation decisions, ultimately leading to improved patient outcomes. Extending this method to other populations, such as large and small gestational-age neonates, should be considered when precise fetal weight percentiles are obtainable.

## Data Availability

The original contributions presented in the study are included in the article/Supplementary Material, further inquiries can be directed to the corresponding author.
